# Distribution of Secretion Systems in the Genus *Legionella* and Its Correlation with Pathogenicity

**DOI:** 10.3389/fmicb.2017.00388

**Published:** 2017-03-14

**Authors:** Tian Qin, Haijian Zhou, Hongyu Ren, Wenbin Liu

**Affiliations:** ^1^State Key Laboratory for Infectious Disease Prevention and Control, National Institute for Communicable Disease Control and Prevention, Chinese Center for Disease Control and PreventionBeijing, China; ^2^Collaborative Innovation Center for Diagnosis and Treatment of Infectious DiseasesHangzhou, China; ^3^Novogene Bioinformatics Technology Co. LtdBeijing, China

**Keywords:** secretion systems, *Legionella*, *L. pneumophila*, whole genome sequence, population structure, pathogenicity evolution

## Abstract

The genus *Legionella* comprises over 60 species, which are important human pathogens. Secretion systems in *Legionella pneumophila* have been studied extensively because of the essential role of protein secretion in bacterial infection. However, there are few reports describing the secretion systems in non-*L. pneumophila* species. In this study, we analyzed the distribution of secretion systems in *L. pneumophila* and 18 species of non-*L. pneumophila* based on whole genome sequences. A total of 74 whole genome sequences from 19 species of *Legionella* were analyzed. Type II and IVB secretion systems were detected in all *Legionella* strains, but the type I secretion systems was restricted to *L. pneumophila.* The type IVA secretion system was randomly distributed among different species. Furthermore, we found the type VI secretion system in three non-*L. pneumophila* strains (*Legionella cherrii* DSM 19213, *Legionella dumoffii* Tex-KL, and *Legionella gormanii* ATCC 33297). In population structure analysis, *L. pneumophila* formed a conservative cluster and was located at the terminal of the evolutionary tree. At the same time, *L. pneumophila*, especially eight clone groups (named MCGG1–MCGG8), showed higher intracellular growth ability than non-*L. pneumophila* species. These results suggest that *L. pneumophila* has acquired additional secretion systems during evolution, resulting in increased pathogenicity.

## Introduction

*Legionella* is the causative agent of Legionnaires’ disease in humans (Fields et al., 2002). It is a Gram-negative facultative intracellular parasite of fresh water amoebae, monocytes and alveolar macrophages (Rogers et al., 1994). *Legionella* is an inhabitant of natural and man-made fresh water environments, where it is present either in a biofilm-associated state or in various protozoa where it can reside and proliferate as an intracellular pathogen (Rogers et al., 1994). Infection rates have increased as a result of industrial settings and the use of technical devices such as air conditioning systems, whirlpools and spas, showers, dental unit water lines and other medical devices, vegetable misters and fountains, which harbor *Legionella* species, combined with an increase in the population of elderly and immuno-compromised individuals (Diederen, 2008).

Transmission of bacteria from the environment to humans occurs via inhalation or aspiration of *Legionella*-containing aerosols (Breiman et al., 1990; Blatt et al., 1993). The genus *Legionella* comprises more than 60 species, among which *Legionella pneumophila* is the major causative agent of human infections, although other species have also been reported to cause disease (Fields et al., 2002; Diederen, 2008). In humans, *Legionella* causes a potentially fatal form of pneumonia, known as legionellosis, as well as a milder form, known as Pontiac fever.

*Legionella* invades, replicates, and survives in human macrophages, mainly by being surrounded by a membrane-bound vacuole that prevents lysosomal degradation (Fields et al., 2002). The mechanism of pathogenicity for *Legionella*, especially the non*-L. pneumophila* species, remains to be elucidated. Protein secretion is a universal process of fundamental importance for various aspects of cell physiology, including the infection of a host organism by a bacterial pathogen. Many Gram-negative pathogens achieve host infection by exporting virulence-associated proteins across one or two cell membranes to the site of action using a wide variety of secretory pathways. These protein secretion systems, which are considered to be important virulence factors in *L. pneumophila*, have been studied extensively. *L. pneumophila* genomes encode multiple protein secretion systems, including the putative type I Lss secretion machinery, type II PilD-dependent Lsp, type IVA Lvh, and type IVB Icm/Dot secretion pathways (De Buck et al., 2007). *L. pneumophila* use these specialized secretion systems to transport effectors and toxins to the extracellular environment or directly into the host cell to modify host physiology and promote interactions (Tseng et al., 2009). Type II and type IVB secretion systems (T4BSS) have been reported to play roles in *L. pneumophila* pathology, containing intracellular infection of protozoans and persistence in the lungs of A/J mice (Liles et al., 1998; Segal and Shuman, 1998; Vogel et al., 1998; Rossier and Cianciotto, 2001; Rossier et al., 2004; De Buck et al., 2007). In this study, we analyzed the genomic sequences of 53 *L. pneumophila* strains and 21 non-*L. pneumophila* strains. The distribution and gene cluster structure of the secretion systems were analyzed, in addition to the population structure and intracellular growth ability to ascertain the relationship between secretion systems and pathogenicity during the evolution of *Legionella*.

## Materials and Methods

### Sequenced Strains and Whole Genome Sequence Analysis

A total of 74 whole genomes of *Legionella* were analyzed in this study. Among them, six isolates were newly sequenced in this study, 44 isolates were sequenced in our previous study (Qin et al., 2016), and the remaining 24 were obtained from the NCBI database. The six newly sequenced strains are *L. bozemanii* strain ATCC 33217, *L. feeleii* strain ATCC 35072, *L. jordanis* strain ATCC 33623, *L. micdadei* strain ATCC 33218, *L. oakridgensis* strain ATCC 33761 and *L. gormanii* strain ATCC 33297. Of the 74 strains, 53 were *L. pneumophila*, comprising 41 serogroup 1 and 12 of other serogroups. The strain ATCC35289# was passed by three generations of strain ATCC35289. The 41 *L. pneumophila* serogroup 1 strains belonged to 26 different sequence-based typing (SBT) types with six SBT groups and three singles were formed among them, showing representative genotypes. As shown in our previous study, the *L. pneumophila* strains belonged to nine minimum core genome groups (MCGG1–MCGG9) (Qin et al., 2016). Another 21 strains belonged to other 19 *Legionella* species. Metadata (species, serogroups, dates and countries of isolation and accession numbers) for the strains included in the genome comparisons are presented in **Supplementary Table S1**.

### DNA Sequencing and Assembly

The genomes were sequenced in Novogene Bioinformatics Technology Co. Ltd, Beijing, China. Bacterial strains were sequenced using an Illumina Hiseq2000 (Illumina Inc., San Diego, CA, USA) with a multiplexed protocol. Paired-end 90 nt-long reads of 500 bp sequencing libraries were generated for six strains. Raw data was processed in a four-step process involving removal of reads with ambiguous bases (5 bp), 20 bp of low quality reads (≤Q20), adapter contamination, and duplicated reads. In total, 100 libraries (500 bp) were obtained with clean paired-end read data. Assembly was performed using SOAPdenovo v1.05 (Li et al., 2008).

### Genes Prediction and Function Annotation

Genes were predicted using Glimmer v3.02 (Delcher et al., 1999). This software predicts start sites and coding regions effectively and achieves good interpolation of hidden Markov models to minimize the frequency of false positive predictions. Function annotation was based on protein sequence analysis and completed by comparing BLAST v2.2.25^1^ results in M8 format to the Kyoto Encyclopedia of Genes and Genomes (KEGG) v59 (Kanehisa et al., 2006), Cluster of Orthologous Groups of proteins (COG) v20090331 (Tatusov et al., 2003; Kanehisa et al., 2004), SwissProt v2011_10_19 (Magrane and Consortium, 2011), NR v2012-02-29, and Gene Ontology (GO) v1.419 databases (Ashburner et al., 2000). Genes were aligned with database sequences to obtain the annotation corresponding to homologs, with the highest quality alignment chosen for the gene annotation.

### Identification of Secretion System Components

We used the ‘tblastn’ algorithm in BLAST (2.2.25 release) (Altschul et al., 1990) to search the *L. pneumophila* genomes for homologs of secretion system components. Perl scripts were developed to select hits with sequence identity >30%, and alignment extended would be implemented, if there’s star or end codon at alignment block boundary areas of 3X bp within 100 bp. Regions of the final alignment with gene coverage homology >40% were considered to be secretion systems components.

### Phylogenetic Analysis

Core-pan analysis of 74 genomes was done and the single-copy orthologous genes were identified. A phylogenetic tree based on concatenated sequences of single-copy orthologous genes was constructed using the maximum-likelihood method with TreeBeST software (Nandi et al., 2010). A phylogenetic tree based on the core genome SNPs was constructed using the neighbor-joining or minimum evolution algorithms in MEGA (version 5.1) (Tamura et al., 2007). Bootstraps were performed with 1,000 replicates. The MEGA program was also used to calculate the *p*-distance (*p* = *n*_d_/*n*) within and between population groups, where *n*_d_ is the number of sites with differences and *n* is the total number of sites.

### Intracellular Growth Assay

The *Legionella* strains were revived from lyophilized isolate. The bacteria were streaked onto buffered charcoal yeast extract (BCYE) agar plates, and one typical colony of each strain was picked up and inoculated onto buffered yeast extract (BYE) broth at 35°C until they reached early stationary phase. Approximately 2 × 10^9^ bacteria were pelleted, resuspended and diluted (1:1,000) in Roswell Park Memorial Institute (RPMI) 1640 tissue culture medium. The bacteria were then added to J774 cells or guinea pig peritoneal macrophages (2 × 10^5^ per well) in 24-well dishes to give a multiplicity of infection (MOI) of approximately 10. The infected cells were incubated at 37°C under 5% CO_2_-air for 1.5 h and then washed three times with PBS to remove extracellular bacteria. To measure bacterial internalization, 1 ml of sterile distilled water was added to the wells to release intracellular bacteria from the host cells. The colony-forming units (CFUs) were determined by plating dilutions on BCYE agar plates. To each of the wells, 0.5 ml of fresh tissue culture medium was added, and the intracellular and extracellular bacteria in each well were combined at 24-h intervals. The total number of CFUs was determined by plating the dilutions onto BCYE agar plates.

The J774 cell monolayers were prepared on cover slips by the same procedures as described above. The cells were infected with *L. pneumophila* philadelphia-1 and the other isolated strains. After 48 h of infection, the infected J774 cells were stained by Gimenez staining and observed under a light microscope.

### Nucleotide Sequence Accession Numbers

This Whole Genome Shotgun project has been deposited at GenBank under the Bioproject ID PRJNA281151, with accession numbers LBAW00000000, LBHK00000000, LBAX00000000, LCUB00000000, LCUA00000000, and LBAY00000000.

## Results and Discussion

**FIGURE 1:**
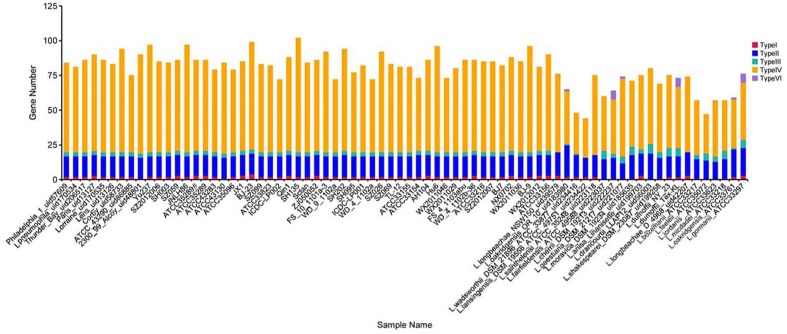
**Number of TnSS functional genes in 74 *Legionella* genomes**. Here, the TnSS contained secretion system protein types I, II, III, IV and VI, collected from the NR, KEGG, COG, POG, GO, PHI, VFDB, ARDB, CAZy databases.

In this study, we analyzed the distribution of *L. pneumophila* and non-*L. pneumophila* secretion system protein types I, II, III, IV and VI by searching the NR, KEGG, COG, POG, GO, PHI, VFDB, ARDB, CAZy databases by using the *Legionella* pan-genome (**Figure 1**). Genes encoding types II and IV secretion system homologs were found in all the strains analyzed, while genes encoding types I and III secretion genes were absent from some non-*L. pneumophila* strains. Additionally, type VI secreted system genes were found in six non-*L. pneumophila* strains.

However, searches conducted using identified secretion system gene cluster sequences in *L. pneumophila* strain Philadelphia 1 revealed genes encoding types I, II, IVA, and IVB secretion system homologs in other genomes, although there were several notable differences. Type II and IVB secretion systems were detected in all *Legionella* strains, but the type I secretion systems (T1SS) was restricted to *L. pneumophila.* The type IVA secretion system (T4ASS) was randomly distributed among different species. Furthermore, we found the type VI secretion system in three non-*L. pneumophila* strains.

### Type I Secretion Systems

**FIGURE 2:**
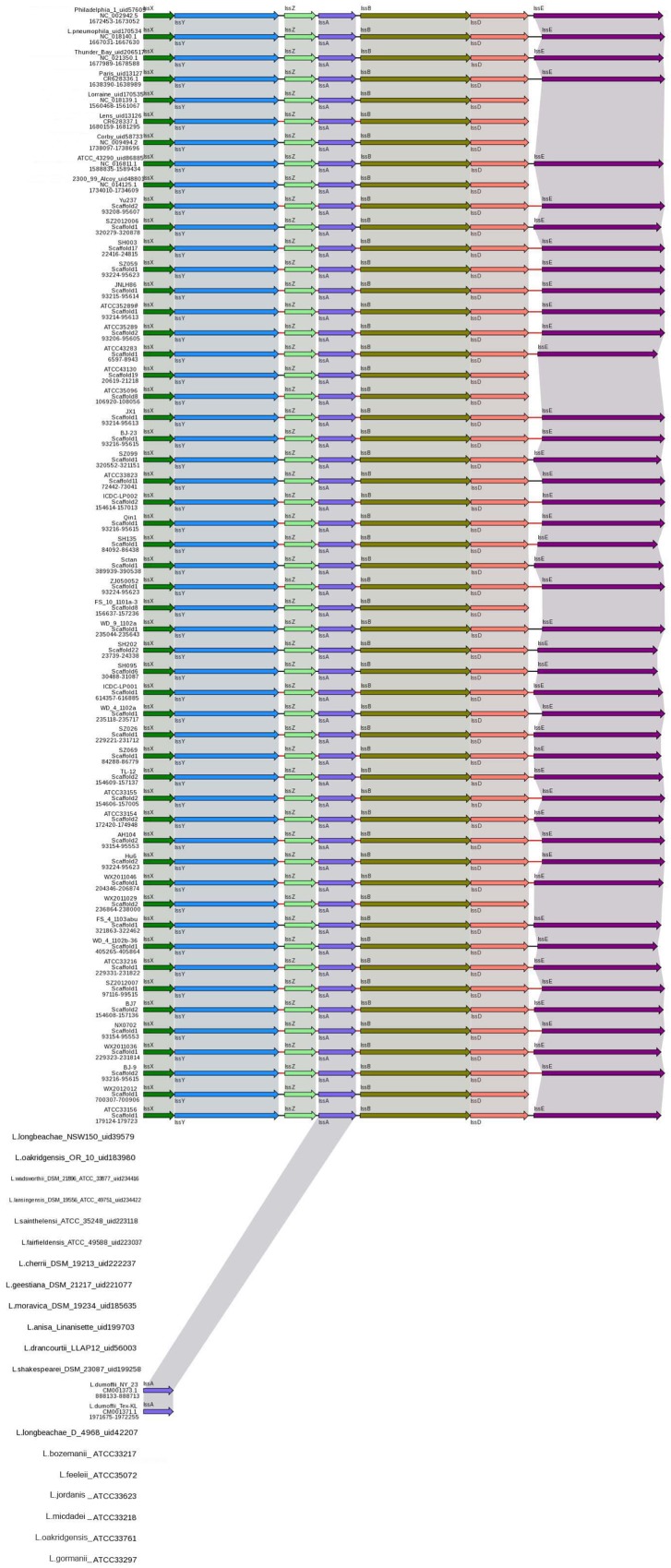
**Structure and distribution of T1SS in 74 *Legionella* genomes**. The box arrows represent open reading frames (ORFs); black lines represent forward scaffolds; red lines represent reverse scaffolds; and genes linked by the same dotted line are homologous.

The T1SS allows secretion of substrates into the extracellular space in a one-step process, without a stable periplasmic intermediate. A T1SS consists of three proteins: an inner-membrane ATPase, known as the ABC (ATP-binding cassette) transporter, a membrane fusion protein spanning the periplasmic space and an outer-membrane protein (Gerlach and Hensel, 2007). In *L. pneumophila*, a putative T1SS designated Lss has been identified. Analysis of the *lss* gene distribution in various *Legionella* strains by Southern blotting suggested that a complete *lss* gene cluster is restricted to *L. pneumophila*, while the *lssD* gene was not detected in non-*L. pneumophila* species (Jacobi and Heuner, 2003). The role of T1SS in *L. pneumophila* virulence has recently been identified and repeats-in-toxin protein RtxA is secreted through an LssB-LssD-TolC-dependent mechanism (Fuche et al., 2015). In this study, all *L. pneumophila* strains contained the *lssXYZABD* locus encoding a putative T1SS. In contrast, the *lssXYZABD* locus was not found in non-*L. pneumophila* species (**Figure 2**), thus indicating that the *lssXYZABD* secretion system is restricted and conserved in *L. pneumophila*.

### Type II Secretion Systems

**FIGURE 3:**
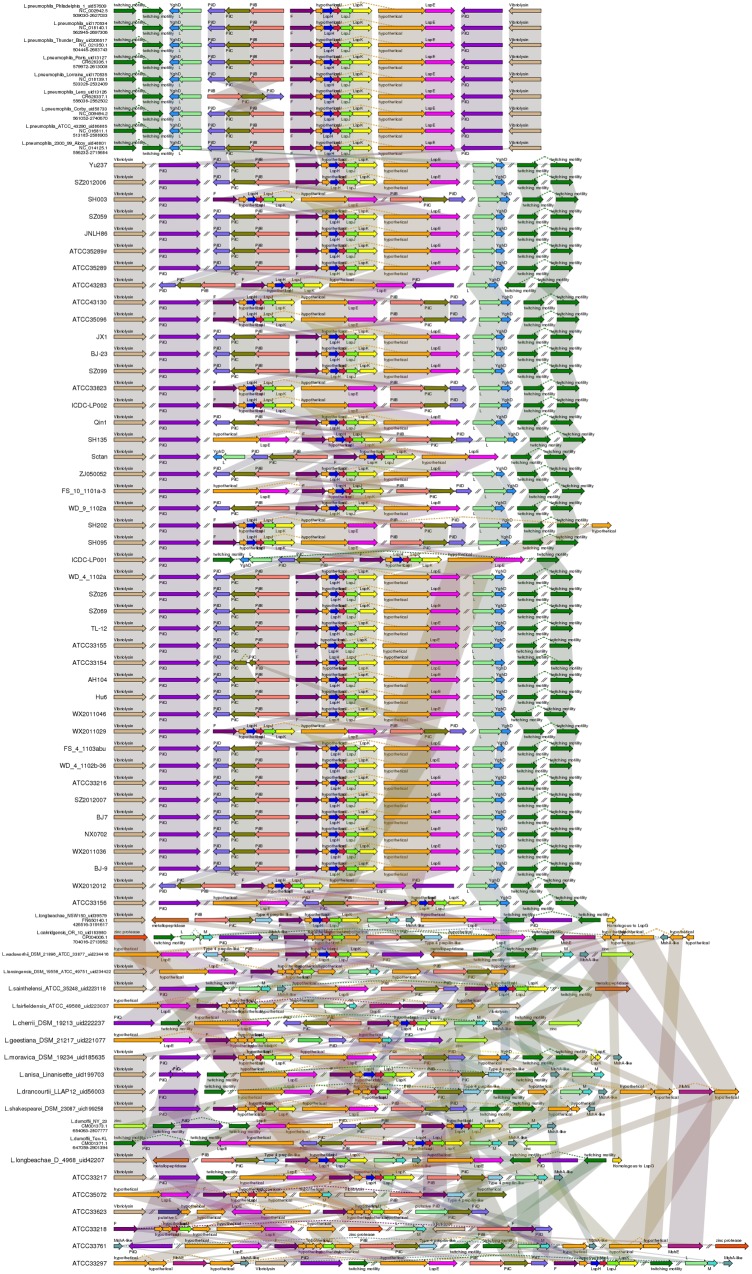
**Structure and distribution of T2SS in 74 *Legionella* genomes**. The box arrows represent ORFs; black lines represent forward scaffolds; red lines represent reverse scaffolds; genes linked by the same dotted line are homologous; and the double forward slash indicates that the genes are located in different scaffolds.

To date, *L. pneumophila* is the only intracellular pathogen known to possess a functional type II secretion system (T2SS) (Cianciotto, 2005). The T2SS system has been shown to promote *L. pneumophila* growth in low temperature environments and is required for biofilm establishment, sliding motility, intracellular infection of protozoans and mammalian macrophages, and persistence in the lungs of A/J mice (Rossier et al., 2004). In a previous study, T2SS genes were identified in several non*-L. pneumophila* species and it was hypothesized that the T2SS occurs throughout the genus *Legionella* (Rossier et al., 2004); this speculation was confirmed by our results. In this study, all analyzed strains, regardless of the species, harbored T2SS genes, containing *pilD* gene and Lsp system (**Figure 3**).

### Type III Secretion System

The type III secretion systems (T3SS) are found in Gram-negative bacteria such as *Salmonella typhimurium*, *Yersinia pestis* and *Pseudomonas syringae*, which interact with both plant and animal hosts (Tseng et al., 2009). In this study, flagellum-encoding genes were found in all *L. pneumophila* and 14 of 21 non*-L. pneumophila* strains. Flagellum-encoding genes were not found in seven non-*L. pneumophila* strains; there were *L. longbeachae* NSW150, *L. wadsworthii* DSM 21896 (ATCC 33877), *L. lansingensis* DSM 19556 (ATCC 49751), *L. sainthelensi* ATCC 35248, *L. longbeachae* D4968, *L. oakridgensis* OR 10 and *L. oakridgensis* ATCC 33761. However, no non-flagellar T3SSs were found.

### Type IV Secretion Systems

The type IV secretion systems (T4SSs) have been grouped into two subclasses; type IVA, which is similar to the *Agrobacterium tumefaciens* Vir system, and type IVB, which is similar to the Tra/Trb bacterial conjugation systems (De Buck et al., 2007). The type IVA Lvh and type IVB Icm/Dot secretion systems are present in *L. pneumophila*.

**FIGURE 4:**
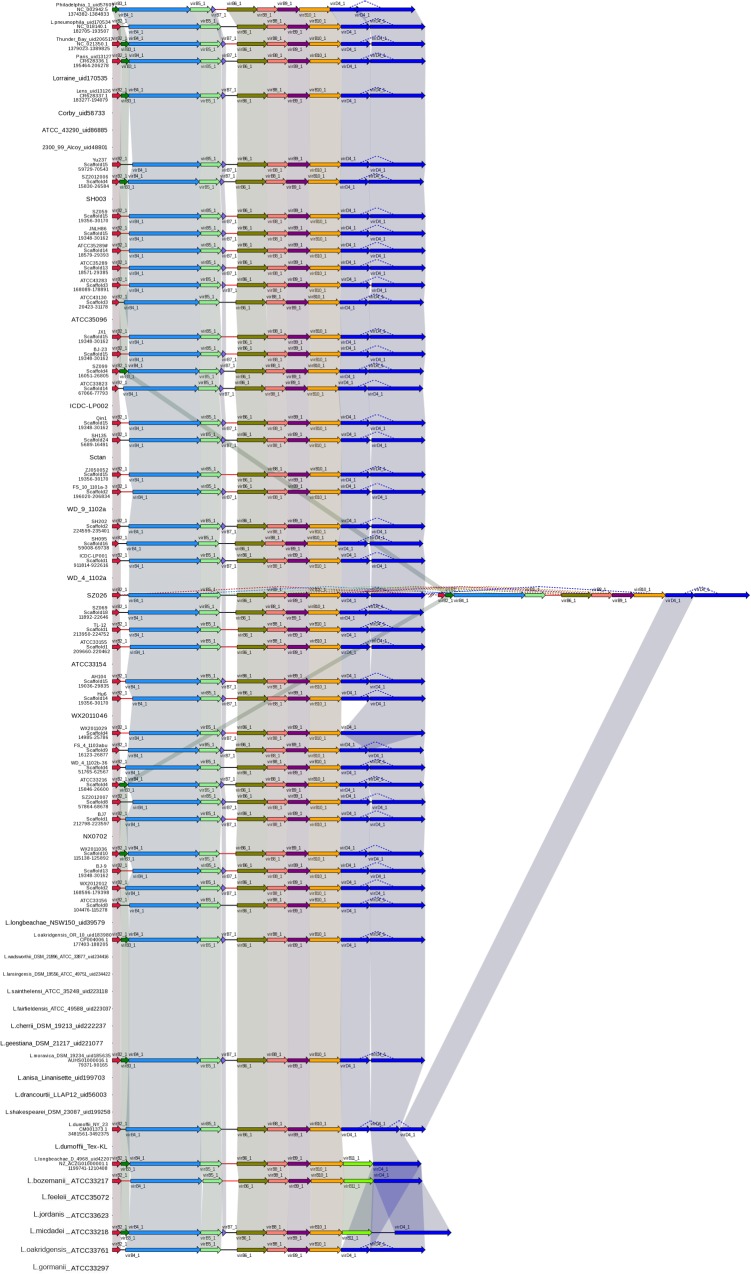
**Structure and distribution of T4ASS in 74 *Legionella* genomes**. The box arrows represent ORFs; black lines represent forward scaffolds; red lines represent reverse scaffolds; genes linked by the same dotted line are homologous; and the double forward slash indicates that the genes are located in different scaffolds.

The gene cluster encoding T4ASS, designated Lvh, is located in a an elevated G*+*C content region of plasmid-like elements, which also contain genes encoding mobility factors and enzymes, such as phage integrases and transposases (Doleans-Jordheim et al., 2006; Glockner et al., 2008). Furthermore, Kozak et al. (2010) hypothesized that these plasmid-like elements can exist in either integrated or excised forms with widespread mobility of the *lvh* region in the genus *Legionella*, thus contributing to interspecies exchange of genetic information; our analyses support this view. In this study, the *Lvh* genes were found in 40 *L. pneumophila* and seven non*-L. pneumophila* strains. The order and sequence of T4ASS Lvh genes are highly conserved among these strains and interestingly, strain SZ026 was found to possess two copies of the *Lvh* secretion system genes (**Figure 4**).

**FIGURE 5:**
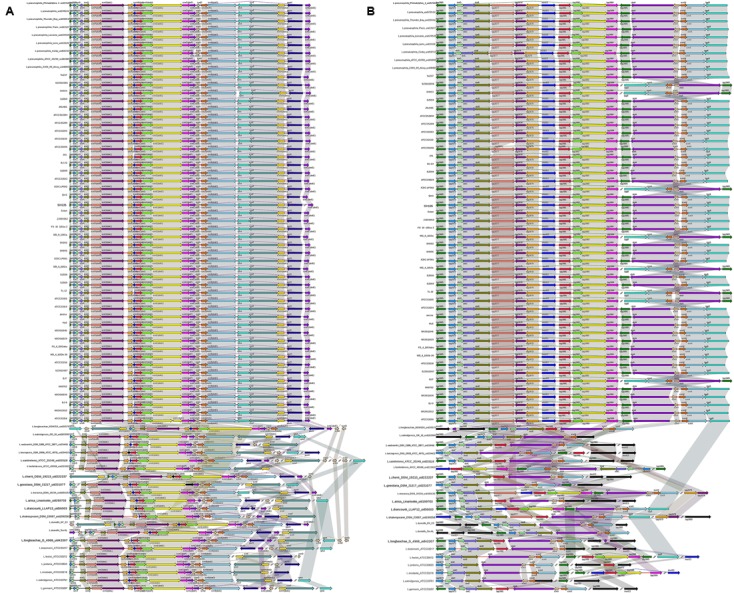
**Structure and distribution of T4BSS region 1** **(A)** and region 2 **(B)** in 74 *Legionella* genomes. The box arrows represent ORFs; black lines represent forward scaffolds; red lines represent reverse scaffolds; genes linked by the same dotted line are homologous; and the double forward slash indicates that the genes are located in different scaffolds.

In *L. pneumophila*, the T4BSS, designated the Icm/Dot secretion system, is encoded by two separate pathogenicity regions on the chromosome. The first region contains 17 genes (icmTSRQ-PONMLKEGCDJBF), while the second region carries icmXWV and dotABCD (Nagai and Kubori, 2011). Our results reveal that the gene organization of both regions is highly conserved in all *L. pneumophila* strains, with relative diversity observed in non*-L. pneumophila* species (**Figure 5**). In region I, all non*-L. pneumophila* strains lack *icmR* and other genes were observed in three non*-L. pneumophila* strains (*L. longbeachae* strain NSW150, *L. cherrii* DSM 19213 and *L. dumoffii* strain NY 23). Furthermore, the *icmF*-*icmH* (*dotU*) genes of region I are separated from the rest of the gene cluster in *L. wadsworthii* DSM 21896 (ATCC 33877), *L. sainthelensi* ATCC 35248, *L. geestiana* DSM 21217, *L. anisa* Linanisette, *L. drancourtii* LLAP12, *L. shakespearei* DSM 23087, *L. longbeachae* D-4968, *L. bozemanii* ATCC 33217, *L. feeleii* ATCC 35072, *L. gormanii* ATCC 33297 (**Figure 5A**). In region II, all strains contained all the genes, except *icmV* in *L. micdadei* strain ATCC 33218; relative diversity was observed among the neighboring Icm/Dot genes (**Figure 5B**). However, in both Icm/Dot regions, the order and orientation of the genes were conserved throughout the genus; this is coincident with the previous findings (Burstein et al., 2016).

### Type VI Secretion System

**FIGURE 6:**
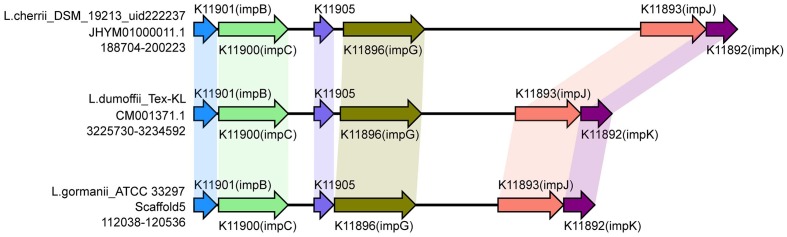
**Structure and distribution of T6SS in 74 *Legionella* genomes**. The box arrows represent ORFs; black lines represent forward scaffolds; red lines represent reverse scaffolds; genes linked by the same dotted line are homologous; and the double forward slash indicates that the genes are located in different scaffolds.

The recently characterized type VI secretion system (T6SS) appears to constitute a phage-tail-spike-like injectisome that has the potential to introduce effector proteins directly into the cytoplasm of host cells. Genes encoding T6SS components are detected in more than a 25% of sequenced bacterial genomes. The T6SS is required for virulence in human, animal and plant pathogens, for efficient root colonization, and may contribute to environmental adaptations, such as biofilm formation, adhesion and cytotoxicity to host cells (Bingle et al., 2008; Cascales, 2008; Filloux et al., 2008; Liu et al., 2008; Shrivastava and Mande, 2008; Wu et al., 2008; Liu et al., 2015). The T6SS in *Legionella* has not been reported previously. In this study, type VI secreted system genes were identified in six strains by searching the NR, KEGG, COG, POG, GO, PHI, VFDB, ARDB, CAZy databases. However, the gene identified in three strains (*L. oakridgensis* OR 10, *L. geestiana* DSM 21217 and *L. oakridgensis* ATCC 33761) was *icmF*, which is a type IV secreted system gene in *Legionella*, and also one of the VI secreted system genes in *Escherichia coli* (de Pace et al., 2011). Another three strains, *Legionella cherrii* DSM 19213, *Legionella dumoffii* Tex-KL, *Legionella gormanii* ATCC 33297, harbored a gene cluster previously identified as T6SS. This gene cluster contains six genes with conserved order (**Figure 6**). This is the first report of the identification and function of T6SS in *Legionella*. It is worth noting that only one of two *L. dumoffii* strains harbors T6SS, suggesting that the presence of T6SS is strain but not species-specific.

### Population Structure and Intracellular Growth Ability

**FIGURE 7:**
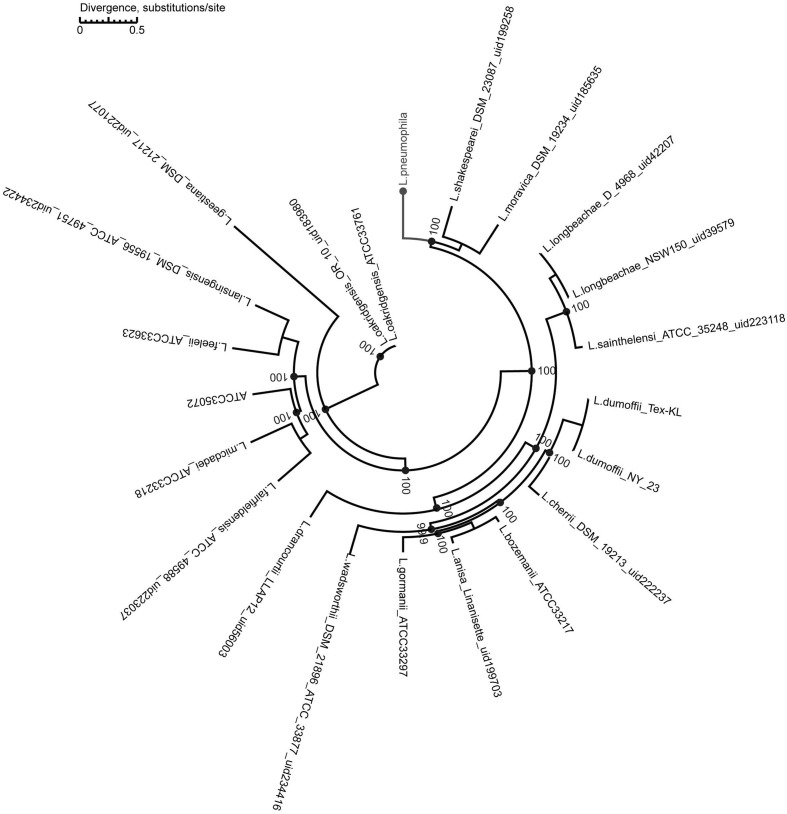
**Maximum-likelihood tree of 74 *Legionella* strains**. This tree was constructed using 300 single-copy orthologous genes identified in 74 *Legionella* genomes.

**FIGURE 8:**
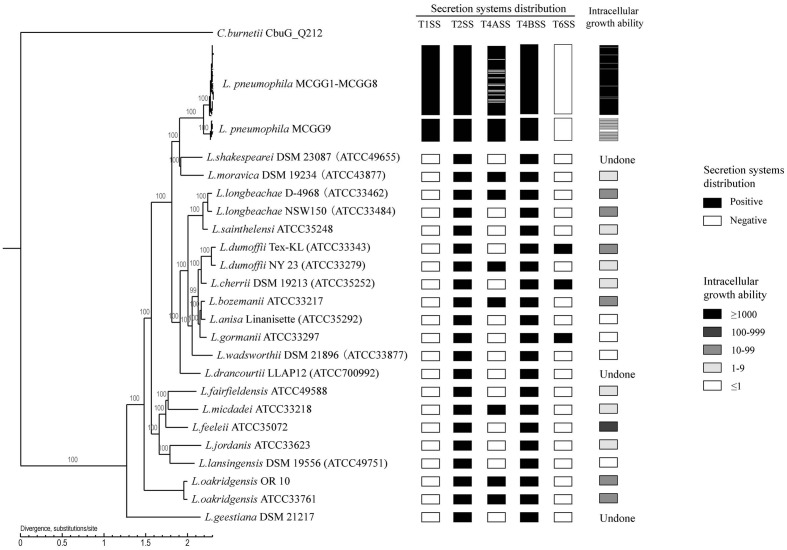
**Rooted phylogenetic tree of 74 *Legionella* strains using 300 single-copy orthologous genes identified in 74 *Legionella* genomes**. *Rickettsia* strain CbuG_Q212 was used as an outgroup. *L. pneumophila* MCGG1–MCGG8 and *L. pneumophila* MCGG9 are reduced to a single cluster each. Secretion system distribution and intracellular growth ability are shown in the left columns. The intracellular growth ability is represented by the fold-increase in bacterial number on day 3 of infection compared with the initial number of bacteria. The two *L. oakridgensis* strains, OR 10 and ATCC 33761, are the same isolate. The genome of OR 10 was obtained from the NCBI database and that of ATCC 33761 was newly sequenced in this study.

We used a maximum-likelihood tree to explore the relationships among our strains, using 300 single-copy orthologous genes identified in 74 *Legionella* genomes (**Figure 7**). Most of the branches in this tree had high supporting bootstrap values, suggesting stable genomic differences among different *Legionella* species. Fifty *L. pneumophila* were clustered as a monophyl, which is consistent with a previous classification system (Qin et al., 2016). *L. moravica* and *L. shakespearei* were more closely related to *L. pneumophila* than to other species, suggesting that these three species had the most recent common ancestor. Among these strains, some strains such as *L. longbeachae* and *L. sainthelensi* strains, and *L. bozemanii* and *L. anisa* strains are more closely related than others. An additional tree constructed using the *Rickettsia* strain CbuG_Q212 as an outgroup (**Figure 8**). While *Legionella geestiana* diverged first from the rest of the species, in this tree, the *L. pneumophila* cluster is more recently derived. This is also supported by the short branches within the *L. pneumophila* cluster. These results suggest that *L. geestiana* may be the first *Legionella* species generated, and that *L. pneumophila* has evolved from other species, most likely *L. moravica* and *L. shakespearei*.

We also tested the intracellular growth ability of *L. pneumophila* and some non*-L. pneumophila* strains. MCGG1–MCGG8 *L. pneumophila* showed significantly higher intracellular growth ability than MCGG9 *L. pneumophila* strains and non*-L. pneumophila* strains. There was no difference in the distribution of secretion systems between MCGG1–MCGG8 and MCGG9 *L. pneumophila* strains; therefore, it can be speculated that the difference in intracellular growth ability is caused by other factors. The differences in distribution of T1SS and the rearrangement and separation of T4BSS region II between *L. pneumophila* and non*-L. pneumophila* strains might account for the difference in intracellular growth ability; however, further studies are required to confirm this speculation.

## Conclusion

Secretion systems are widely distributed in genus *Legionella*. In this study, all the *Legionella* strains analyzed harbor complete T2SS and T4BSS gene clusters; however, T1SS is restricted to *L. pneumophila*. T4ASSs exist in some of the *L. pneumophila* and non-*L. pneumophila* species. Three non-*L. pneumophila* strains harbor T6SS. Population structure analysis revealed that *L. pneumophila* forms a conservative cluster and located on the terminal of the evolutionary tree. *L. pneumophila* also showed higher intracellular growth ability than non-*L. pneumophila* species. The limitation of this study is only 20 species were analyzed, as well as there are more than 60 species of *Legionella*. However, these results suggest that *L. pneumophila* has acquired additional secretion systems during evolution, resulting in increased pathogenicity.

## Author Contributions

TQ and HZ designed experiments. TQ and HR performed experiments. TQ, HZ, and WL analyzed data. TQ and WL contributed reagents, materials, and analysis tools. TQ and HZ wrote this manuscript. All authors reviewed the manuscript.

## Conflict of Interest Statement

The authors declare that the research was conducted in the absence of any commercial or financial relationships that could be construed as a potential conflict of interest.

## Footnotes

1 http://blast.ncbi.nlm.nih.gov/Blast.cgi

## Supplementary Material

TABLE S1 **Metadata for the strains included in the genome comparisons in this study**.
